# Preserving Genome Integrity: The DdrA Protein of Deinococcus radiodurans R1

**DOI:** 10.1371/journal.pbio.0020304

**Published:** 2004-09-07

**Authors:** Dennis R Harris, Masashi Tanaka, Sergei V Saveliev, Edmond Jolivet, Ashlee M Earl, Michael M Cox, John R Battista

**Affiliations:** **1**Department of Biochemistry, University of WisconsinMadison, WisconsinUnited States of America; **2**Department of Biological Sciences, Louisiana State University and A & M CollegeBaton Rouge, LouisianaUnited States of America

## Abstract

The bacterium Deinococcus radiodurans can withstand extraordinary levels of ionizing radiation, reflecting an equally extraordinary capacity for DNA repair. The hypothetical gene product DR0423 has been implicated in the recovery of this organism from DNA damage, indicating that this protein is a novel component of the D. radiodurans DNA repair system. DR0423 is a homologue of the eukaryotic Rad52 protein. Following exposure to ionizing radiation, DR0423 expression is induced relative to an untreated control, and strains carrying a deletion of the DR0423 gene exhibit increased sensitivity to ionizing radiation. When recovering from ionizing-radiation-induced DNA damage in the absence of nutrients, wild-type D. radiodurans reassembles its genome while the mutant lacking DR0423 function does not. In vitro, the purified DR0423 protein binds to single-stranded DNA with an apparent affinity for 3′ ends, and protects those ends from nuclease degradation. We propose that DR0423 is part of a DNA end-protection system that helps to preserve genome integrity following exposure to ionizing radiation. We designate the DR0423 protein as DNA damage response A protein.

## Abstract 2

Deinococcus radiodurans is able to repair radiation induced genome damage by virtue of a homologue of the eukaryotic Rad52 protein, which preserves genome integrity by protecting DNA ends

## Introduction

Deinococcus radiodurans is a non-spore-forming bacterium notable for its capacity to tolerate exposure to ionizing radiation (Battista and Rainey 2001). The D_37_ dose for D. radiodurans R1 is approximately 6,500 Gy, at least 200-fold higher than the D_37_ dose of Escherichia coli cultures irradiated under the same conditions. The energy deposited by 6,500-Gy γ radiation should introduce thousands of DNA lesions, including hundreds of double-stranded breaks (Smith et al. 1992). The mechanisms responsible for this species' resilience are poorly described, and recent analyses of DNA-damage-induced changes in the proteome (Lipton et al. 2002) and transcriptome (Liu et al. 2003) of D. radiodurans cultures have done little to improve our understanding of *D. radiodurans'* radioresistance (Edwards and Battista 2003; Narumi 2003).

For most species, the intracellular generation of strand breaks has lethal consequences; exposed free ends serve as substrates for intracellular exonucleases that degrade the genome. However, in D. radiodurans the presence of strand breaks does not result in a catastrophic loss of genetic information (Dean et al. 1966; Lett et al. 1967; Vukovic-Nagy et al. 1974). Instead, this species appears to have the ability to control DNA degradation postirradiation by synthesizing proteins that prevent extensive digestion of the genome, and it has been suggested that the DNA degradation observed in this species is an integral part of the process of DNA repair, generating single-stranded DNA that promotes homologous recombination and restitution of the damaged genome (Battista et al. 1999).

When D. radiodurans is exposed to a high dose of ionizing radiation, a number of genes are induced that lack readily identifiable homologues among known prokaryotic proteins (Liu et al. 2003; Tanaka et al. 2004). Among these is the gene designated DR0423. This locus is one of the most highly induced genes in *Deinococcus* following γ-irradiation, with expression increasing 20- to 30-fold relative to an untreated control. Although originally annotated as a “hypothetical” protein (White et al. 1999), a more detailed analysis (Iyer et al. 2002) has identified an evolutionary relationship between DR0423p and the important eukaryotic recombination protein Rad52. Rad52 is part of a larger family of proteins exhibiting structural similarity but little sequence homology, including the prokaryotic Redβ, RecT, and Erf proteins (Passy et al. 1999; Iyer et al. 2002).

In this report, we provide evidence for a DNA end-protection system in D. radiodurans and characterize the DR0423 protein as a component of that system. Our studies suggest that DNA end protection might be particularly important to this species in the context of long-term survival during desiccation and recovery in a nutrient-poor environment.

## Results

### Transcripts Corresponding to the Coding Sequence Designated DR0423 Increase in Response to Sublethal Doses of Ionizing Radiation

During the course of microarray studies intended to establish which R1 loci respond to ionizing radiation, it was noted that transcripts of DR0423 were among the mostly highly induced (Tanaka et al. 2004). As an independent confirmation of these microarray results, the expression of this gene was monitored using quantitative real-time PCR. Total RNA was isolated from exponential-phase cultures of R1 immediately after and at 30 and 60 min following exposure to 3,000-Gy ionizing radiation. Changes in transcript abundance for the *recA* (DR2340), *gap* (DR1343), and DR0423 genes were determined as previously described (Earl et al. 2002a). The results of these analyses are listed in Table 1. Consistent with previous results, levels of *recA* transcript increased postirradiation (Narumi et al. 2001; Bonacossa de Almeida et al. 2002; Satoh et al. 2002), whereas *gap* induction remained unchanged (Earl et al. 2002a). The *gap* gene encodes glyceraldehyde 3-phosphate dehydrogenase and does not respond to DNA damage. Within one-half hour postirradiation, levels of DR0423 transcript increased 20- to 30-fold, suggesting that DR0423p may be a previously unrecognized component of the cell's defense against ionizing-radiation-induced damage.

**Table 1:**
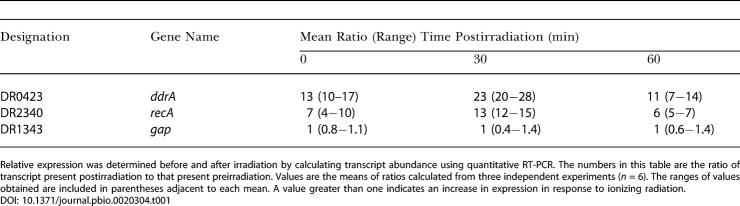
Relative Expression of the *ddrA*, *recA,* and *gap* Genes of D. radiodurans R1 following Exposure to 3,000-Gy Ionizing Radiation

Relative expression was determined before and after irradiation by calculating transcript abundance using quantitative RT-PCR. The numbers in this table are the ratio of transcript present postirradiation to that present preirradiation. Values are the means of ratios calculated from three independent experiments (*n* = 6). The ranges of values obtained are included in parentheses adjacent to each mean. A value greater than one indicates an increase in expression in response to ionizing radiation

### Deletion of DR0423 Sensitizes D. radiodurans R1 to Ionizing Radiation and Mitomycin C

**Figure 1:**
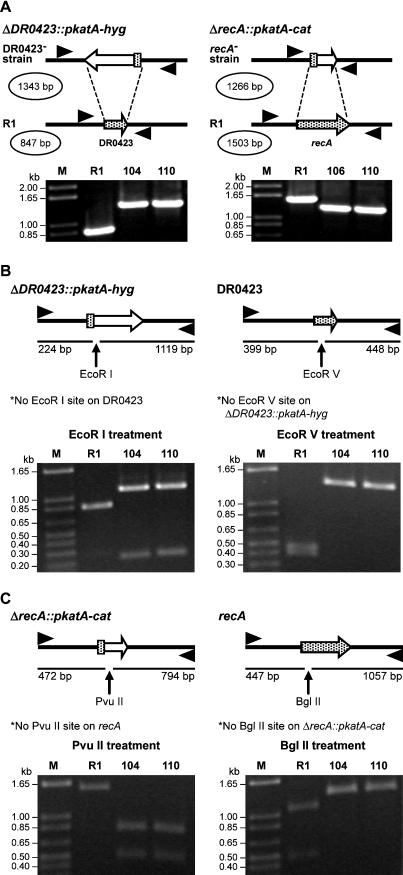
Verification of Gene Deletions (A) Verification of *ddrA* and *recA* gene deletions by PCR analysis. Purified PCR fragments were amplified from the genomic DNA of strains R1, TNK104, TNK106, and TNK110 using primers that flank the coding sequences for *ddrA* and *recA.* Products were separated on a 0.8% agarose gel to establish whether the fragment size corresponded to the gene-replacement cassette. The left panel depicts the replacement of *ddrA* in TNK104 and TNK110. The right panel depicts the replacement of *recA* in TNK106 and TNK110. Expected sizes of the wild-type and mutant sequences are given in the figure above each image of the agarose gel. (B) Verification of the *ddrA* gene deletion by restriction analysis of purified PCR products. Purified PCR fragments were amplified from the genomic DNA of strains R1, TNK104, and TNK110, using primers that flank the coding sequences for *ddrA.* Products were restricted with EcoR1 (left panel) and EcoRV (right panel) to verify their identity. Products were separated on a 0.8% agarose gel to establish whether the restriction fragment corresponded with the expected sizes as illustrated in the figure above each image of the agarose gel. (C) Verification of the *recA* gene deletion by restriction analysis of purified PCR products. Purified PCR fragments were amplified from the genomic DNA of strains R1, TNK106, and TNK110, using primers that flank the coding sequences for *recA.* Products were restricted with PvuII (left panel) and BglII (right panel) to verify their identity. Products were separated on a 0.8% agarose gel to establish whether the restriction fragment corresponded with expected sizes as illustrated in the figure above each gel.

**Figure 2:**
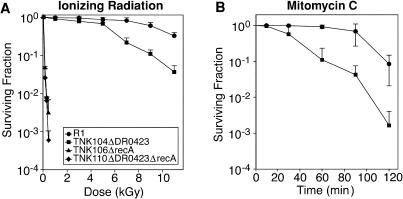
DNA Damage Sensitivity of D. radiodurans Cells Lacking DdrA Function (A) Representative survival curves for D. radiodurans strain TNK104 *ΔddrA* (squares) and D. radiodurans R1 (circles) following exposure to γ radiation. Survival of strains; values are the mean ± standard deviation of three independent experiments; *n* = 9. (B) Representative survival curves for D. radiodurans strain TNK104 *ΔddrA* (squares) and D. radiodurans R1 (circles) following exposure to mitomycin C. Values are the mean ± standard deviation of three independent experiments; *n* = 9.

The DR0423 gene was inactivated by deletion in D. radiodurans R1, as described elsewhere (Funayama et al. 1999), and the resulting strain designated TNK104. Confirmation of the gene deletion is provided in Figure 1. Deletion of DR0423 does not alter the growth rate of the culture (approximately 1.5-h doubling time), or decrease the efficiency of natural transformation (approximately 5 × 10^−5^ rifampicin-resistant transformants per colony-forming unit ) relative to R1, indicating that DR0423p is not essential for the processes of DNA replication or homologous recombination. To establish whether DR0423p was necessary for DNA damage tolerance, TNK104 was evaluated for its ability to survive ionizing radiation and mitomycin C. Aliquots of exponential-phase cultures were exposed to these DNA-damaging agents. TNK104 exhibits increased sensitivity to both agents relative to the wild-type R1 strain (Figure 2), but cultures only displayed significant ionizing radiation sensitivity at doses in excess of 5,000 Gy. Since expression of DR0423 increases in response to ionizing radiation, and its gene product contributes to the DNA damage resistance of this species, we have chosen to designate this gene as *DNA damage response A (ddrA)*.

### A *ddrA recA* Double Mutant Is More Sensitive to Ionizing Radiation Than Either Single Mutant

**Figure 3:**
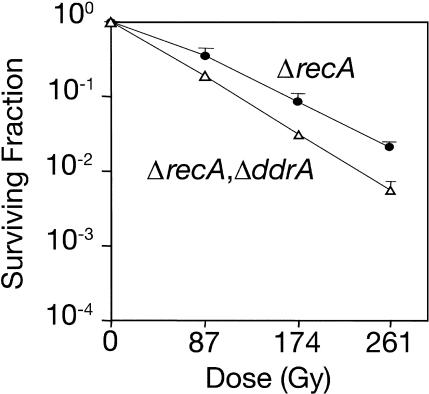
DdrA Functions in a RecA-Independent DNA Repair Process Representative survival curves for D. radiodurans strains TNK106 *ΔrecA* (closed circles) and TNK110 *ΔddrA ΔrecA* (open triangles) following exposure to lower levels of γ radiation. All values are the mean ± standard deviation of three independent experiments; *n* = 9.

The *recA* gene was deleted from R1 and TNK104 (see Figure 1B and 1C), resulting in strains TNK106 *(ΔrecA)* and TNK110 *(ΔrecA, ΔddrA),* respectively. Deinococcal strains lacking *recA* function are considered the most ionizing-radiation-sensitive strains described for this species (Moseley and Copland 1975; Gutman et al. 1994). However, as indicated in Figure 3, TNK110 is 3- to 5-fold more sensitive to ionizing radiation than the *ΔrecA* strain, indicating that DNA damage response A protein (DdrA), at least in part, contributes to *D. radiodurans'* survival by a mechanism that is independent of RecA function.

### Evidence That the DdrA Protein Contributes to Genome Restitution

To determine if loss of DdrA affected genome restitution and stability postirradiation, we followed the recovery of cultures of R1 and TNK104 following a 5,000-Gy dose of γ radiation. Initially, exponential-phase cultures were harvested, suspended in 10 mM MgSO_4_, and irradiated. No carbon source was added. Restoration of the genome was monitored by pulsed-field gel electrophoresis, and aliquots retrieved from the recovering cultures were used to determine viability. Cultures were left in this medium and sampled at 24-h intervals over a 120-h time course.

**Figure 4:**
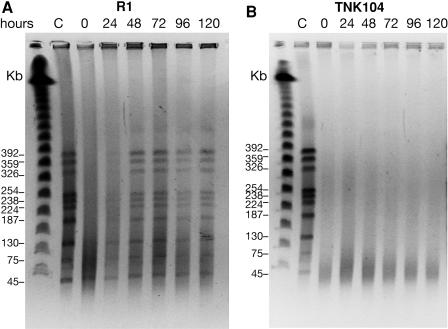
Genome Recovery in the Absence of Nutrients Depends on DdrA (A) Pulsed-field gel electrophoresis analyses of D. radiodurans strain RI recovery over a 120 h time course in 10 mM MgSO_4_ following 5,000-Gy γ radiation. (B) Pulsed-field gel electrophoresis analyses of D. radiodurans strain TNK104 (*ΔddrA*) recovery following 5,000-Gy γ radiation.

**Figure 5:**
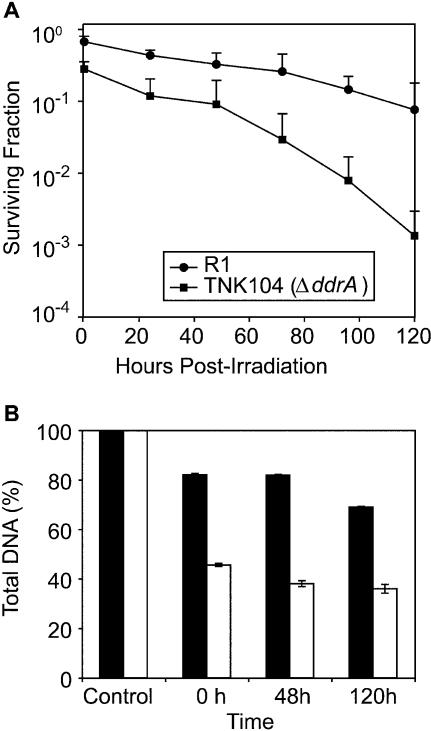
DdrA Protein Effects on In Vivo Survival and Genome Preservation following Exposure to Ionizing Radiation in the Absence of Nutrients (A) Survival of D. radiodurans R1 and TNK104 cultures held in 10 mM MgSO_4_ for 120 h following exposure to 5,000-Gy γ radiation. Samples were obtained at 24-h intervals. All values are the mean ± standard deviation of three independent experiments; *n* = 9 (B) Changes in DNA content in cultures of R1 and TNK104 recovering from exposure to 5,000-Gy γ radiation in MgSO_4_. The DNA concentration at each time point is expressed as a percentage of that present in each strain prior to irradiation.

The gel depicted in Figure 4 illustrates the reassembly of the genomes of irradiated R1 cells. There are 11 NotI sites in the D. radiodurans genome, and when restricted, most of the resulting fragments can be separated by pulsed-field gel electrophoresis as seen in the lane (C) corresponding to the unirradiated control. Immediately after irradiation, the introduction of DNA double-stranded breaks results in the disappearance of the higher molecular weight NotI fragments, but the pattern of fragments is restored in 24–48 h, indicating that R1 is repairing double-stranded breaks under these conditions, in spite of the absence of nutrients. This pattern persists throughout the rest of the time course, indicating that once reformed the genome is stable. Despite genome restitution, R1 cultures held in MgSO_4_ are not as proficient at recovering from ionizing-radiation-induced damage as cultures that are allowed to recover in rich medium (Figure 4; data not shown). Even if plated immediately after exposure, R1 cultures suspended in MgSO_4_ exhibit a modest 2-fold reduction in viability when exposed to 5,000-Gy γ radiation relative to R1 cultures irradiated in rich medium (see Figure 2). The longer the culture is held in MgSO_4_ (Figure 5), the greater the reduction in viability. After 120 h, approximately 10% of the irradiated R1 population remains viable. In comparison, 80% of an unirradiated exponential-phase population of R1 is viable when kept in 10 mM MgSO_4_ for 5 d (data not shown).

Irradiated TNK104 cultures are significantly more vulnerable to ionizing radiation during a prolonged incubation in MgSO_4_ (Figure 5A). TNK104 cultures exhibit only 0.1% survival after 120 h, a 100-fold reduction relative to identically treated R1 cultures. Also, in sharp contrast to the R1 cultures (see Figure 4A), there is no evidence of genome reassembly in the TNK104 cells over this time course (see Figure 4B), suggesting that failure to reassemble the genome contributes to the lower viability observed in TNK104 cultures.

We directly examined the influence of DdrA on the fate of genomic DNA (see Figure 5B) by monitoring changes in DNA content as the cultures of R1 and TNK104 recovered from exposure to 5,000 Gy in MgSO_4_. An aliquot of each unirradiated culture was isolated and total DNA concentration for 10^6^ cfu calculated. Following irradiation, the DNA content of a volume corresponding to the original 10^6^ cfu of each culture was determined. The DNA concentration at each time point in Figure 5B is expressed as a percentage of that present in each strain prior to irradiation. Immediately after irradiation, the genomic DNA in the R1 culture was reduced by approximately 18%, a value consistent with previous findings (Dean et al. 1966; Lett et al. 1967; Vukovic-Nagy et al. 1974) that indicate that 20%–25% of the genomic DNA of D. radiodurans will be degraded and expelled from the cell following exposure to 5,000-Gy γ radiation. In contrast, genomic DNA degradation in the strain lacking DdrA approached 55%. Thus, the presence of DdrA has a greater than 3-fold effect on the preservation of genomic DNA during early times after irradiation. In the succeeding 120 h, the R1 genomic DNA was reduced by a total of 31%, while the loss of genomic DNA increased to 64% in TNK104. These results suggest that DdrA has a direct effect on the preservation of genomic DNA following extreme insults.

We also examined genome restitution in a rich medium (TGY broth). Consistent with the survival curve depicted in Figure 2, we found that when TNK104 cells are exposed to 5,000 Gy their genomes reassemble with kinetics identical to those of the wild-type R1 culture (Grimsley et al. 1991; Mattimore and Battista 1996); the genome reforms in less than 6 h (data not shown). Thus, DdrA appears to contribute to genome reconstruction in D. radiodurans following irradiation, but this role was only obvious in cultures suspended in MgSO_4_. There could be at least two explanations for this observation. First, the action of DdrA may overlap with the activity of at least one other protein, and while each redundant activity is functional in rich medium, only DdrA is functional in cultures held in MgSO_4_. Alternatively, the primary role of DdrA could be the passive protection of exposed 3′ DNA ends at the sites of DNA strand breaks. Under conditions with limiting nutrient availability, DdrA could contribute to genome restitution simply by preventing the massive genomic degradation evident in Figure 5B. In a rich medium, active DNA repair may render DdrA-mediated DNA protection less important.

### The Purified DdrA Protein Binds the 3′ Ends of Single-Stranded DNA and Protects Them from Digestion by an Exonuclease

**Figure 6:**
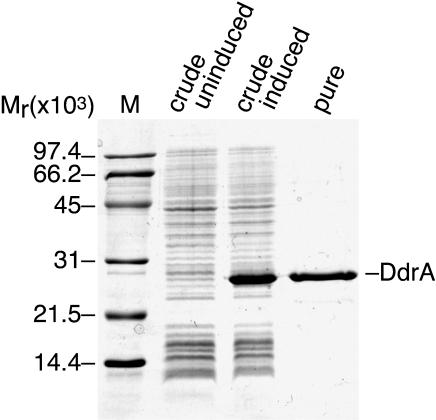
Purification of the DdrA Protein The first lane contains molecular weight markers. The second and third lanes contain crude extracts from E. coli strain pEAW298 (DdrA overproducer) in which the *ddrA* gene is uninduced or induced, respectively. The final lane contains purified DdrA protein.

The *ddrA* gene was cloned and expressed in *E. coli,* and the protein was purified to homogeneity (Figure 6). The identity of the purified protein was confirmed by N-terminal sequencing and mass spectrometry. The deduced N-terminal sequence was MKLSDV, matching the predicted sequence of the first six amino acids perfectly (with the initiating methionine retained). The measured mass of the protein was 23,012.8 ± 3.46 Da, in good agreement with the 23,003.38 Da predicted. In two gel-filtration experiments using a Sephacryl S300 column calibrated with molecular weight standards, DdrA eluted as a sharp peak with an apparent mass in the two different trials of 218 and 190 kDa (data not shown). These results suggest that DdrA is an oligomer in solution with 8–10 subunits. Whereas these results are preliminary, Rad52 protein and other members of this family function as large oligomeric rings (Passy et al. 1999; Iyer et al. 2002; Singleton et al. 2002)

**Figure 7:**
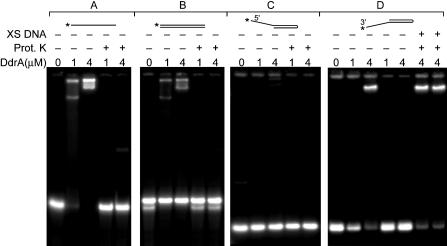
DdrA Protein Binds to Single-Stranded DNA with Free 3′ Ends Four sets of EMSAs are presented, with the gels and electrophoresis conditions carefully matched. DNA substrate concentrations are 0.7 nM in each case, reported as total molecules. In each set, the first three lanes show the effects of the indicated concentration of DdrA protein. The fourth and fifth lanes are identical to the second and third lanes, respectively, except that they are treated with proteinase K to demonstrate that the DNA has not been altered. In set D, the sixth and seventh lanes are identical to the third lane (with 4 μM DdrA protein), except that they have been challenged with a 1,000-fold or 2,000-fold excess of unlabeled oligo with a 5′ extension, respectively. The unlabeled challenge oligo is the same as that used in reaction set C. (A) Single-stranded oligonucleotides (51 nt in length), labeled on the 5′ end. (B) 5′ end–labeled duplex DNA fragments (51 bp). (C) 5′ end–labeled oligonucleotide, with a self-complementary sequence leading to the formation of an 18-bp hairpin and a 15-nt 5′ single-stranded extension. (D) 3′ end–labeled oligonucleotide, with a self-complementary sequence leading to the formation of an 18-bp hairpin and a 16-nt 3′ single-stranded extension. The sequences of the single-stranded extensions in the oligos used in sets C and D are matched, except that an extra adenosine residue has been added to the oligo used in set D during the labeling process. Note that in set B, only the lower substrate band (unannealed oligonucleotides) is bound by DdrA, and the migration of the resulting complexes is identical to that shown in set A.

DdrA exhibited no ATPase, helicase, recombinase, or nuclease activity (data not shown). However, it bound to single-stranded DNA as determined by an electrophoretic mobility-shift assay (EMSA) (Figure 7). Binding to duplex DNA depended on the presence of a 3′ single-stranded extension at one end (Figure 7), indicating that the protein has some affinity for a free 3′ end in single-stranded DNA. This binding was not disrupted by a challenge with a 1,000- to 2,000-fold excess of a duplex oligonucleotide with a 5′ single-stranded extension (Figure 7).

**Figure 8:**
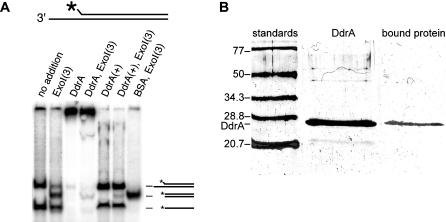
DdrA Protein Protects 3′ Ends from Degradation by Exonuclease I (A) This set of reactions uses the labeled duplex DNA illustrated. The oligos annealed to form this DNA are 51 and 37 nt in length and pair so as to leave a 14-nt 3′ extension. The shorter DNA is 5′ end–labeled. The first lane contains unreacted DNA, showing both the annealed duplex and the unannealed single-stranded DNA. The second lane shows the DNA after treatment with 3 units of exonuclease I for 7 min in a 15-μl reaction mixture. Note that the duplex DNA in the upper band has been shortened by removal of the single-stranded extension. In lanes 3 and 4, the DdrA protein (4 μM) has been incubated with the DNA, without and with the 3 units of exonuclease I, respectively. The DNA is bound by DdrA and shifted to the top of the gel. The reactions shown in lanes 5 and 6 are identical to those in lanes 3 and 4, but with SDS and proteinase K added to disrupt the DdrA–DNA complexes and reveal that the DNA has been minimally affected by exonuclease I. The final lane shows another reaction of the DNA with 3 units of exonuclease I, in the presence of 4 μM bovine serum albumin. Exonuclease I degrades single-stranded DNA in the 3′ to 5′ direction. (B) The protein bound to the duplex DNA is DdrA. The reaction of lane 3 in (A) was scaled up and the protein–DNA complex excised from the gel as described in Materials and Methods. The protein in this complex was subjected to electrophoresis on an SDS-polyacrylamide gel, shown here (lane 3). The control lanes contained prestained protein standards (lane 1) and purified DdrA protein (lane 2). The gel-extracted protein comigrated with DdrA.

DdrA also protected the single-stranded DNA from degradation by exonuclease I from *E. coli,* which digests single-stranded DNA from the 3′ end (Figure 8). The DNA binding trials shown in Figure 8A were scaled up, and the bound species was cut out of a preparative gel. The extracted protein comigrated with DdrA protein on a sodium dodecyl sulfate (SDS)-polyacrylamide gel (Figure 8B), providing further confidence that the binding is due to DdrA and not a minor contaminant in the DdrA protein preparation. These results suggest that D. radiodurans possesses a novel DNA end-protection system and that DdrA is a component of that system.

The eukaryotic Rad52 protein has a single-stranded annealing activity that may be important to its in vivo function (Mortensen et al. 1996; Sugiyama et al. 1998). We carried out several tests to determine if the DdrA protein had a similar annealing activity. In multiple trials using oligonucleotides of 30 and 51 nucleotides (nt) in length, no DNA strand annealing activity was detected over a range of DdrA concentrations and conditions (data not shown).

## Discussion

The extraordinary resistance of D. radiodurans to DNA damage arose not as an adaptation to high levels of radiation, but rather as a response to desiccation (Mattimore and Battista 1996). In an arid environment, dormant D. radiodurans cells would gradually accumulate DNA lesions of all kinds, including strand breaks. Since DNA repair is highly reliant on metabolic energy, and appropriate nutrients cannot be assured upon rehydration, it is not unreasonable to expect that this species possesses a means to efficiently repair accumulated damage that minimizes energy use. In this context, mechanisms must have evolved to maintain the genome and protect it from unnecessary degradation by nucleases and other agents. In this study we have identified functions associated with a “hypothetical” protein encoded by D. radiodurans R1 that contributes to this species' capacity to tolerate exposure to ionizing radiation and mitomycin C. We propose that the DR0423 protein, which we have designated DdrA, is part of a DNA end-protection system. Induced in response to the appearance of strand breaks generated by ionizing radiation (or subsequent to desiccation), DdrA would cap the strand breaks and help stabilize the genome until such time as conditions were more amenable to systematic DNA repair.

The results we have obtained both in vivo and in vitro are consistent with this hypothesis. When the *ddrA* gene is deleted from R1, an otherwise wild-type cell becomes more sensitive to DNA-damaging agents (see Figure 2). We show that DdrA has at least two activities: DdrA contributes to genome restitution following irradiation (see Figure 4), and purified DdrA binds the 3′ ends of single-stranded DNA and protects those ends from digestion by exonucleases (see Figures 7 and 8). Notably, the effects of a *ddrA* deletion are amplified if nutrients are not provided after exposure to ionizing radiation, and cells held this way for 5 d display a 100-fold reduction in viability relative to the wild-type cells (see Figure 5). In these nutrient-poor conditions, cells lacking DdrA protein do not restore their chromosomes. Instead, the chromosomes are degraded extensively.

Even though the R1 strain was able to restore its genome following irradiation and incubation in 10 mM MgSO_4_, there was no evidence of genome reassembly in similarly treated cultures of TNK104, the *ΔddrA* derivative of R1 (see Figure 4). This result indicates that DdrA plays a qualified role in genome restitution. Clearly the protein is necessary for this process in cells held in MgSO_4_, and we suggest that TNK104's inability to reconstitute its genome under these conditions is likely to be related to the DNA degradation that is observed in this strain following irradiation (see Figure 5B).

DdrA is not needed if cells are allowed to recover in a nutrient-rich medium (see Figure 2). This suggests that the function that DdrA mediates in genome restitution is either redundant or unnecessary when other repair processes are robust. If there is a protein with a redundant activity, it is evident only in rich medium. We do not know the identity of the redundant component, or understand why it is not functional in MgSO_4_. Since DdrA binds the 3′ ends of single-stranded DNA, we presume that this protein either has the same activity or is rendered unnecessary by a compensating activity possible only in a nutrient-rich environment (such as DNA synthesis to counter exonucleolytic degradation). If, instead, DdrA is part of a passive DNA protection system, this system may be critical under conditions in which active (energy-requiring) DNA repair is not possible, such as when cells are desiccated or held in a nutrient-free medium. DdrA may not be as important in a nutrient-rich environment, where active DNA synthesis and other DNA repair processes may compensate for the loss of DNA end protection.

The increased sensitivity observed in TNK110 *(ΔrecA ΔddrA)* relative to TNK106 *(ΔrecA)* indicates that DdrA participates in a process that complements RecA-mediated survival mechanisms (see Figure 3), rescuing some irradiated cells even in the absence of RecA function. Since DdrA is distantly but specifically related to the Rad52 family of eukaryotic proteins, as well as a family of phage-associated proteins that mediate single-stranded annealing (Iyer et al. 2002), we speculate that DdrA could be a component of a single-stranded annealing system that functions simultaneously with RecA-dependent homologous recombination. This possibility is consistent with an earlier report by Daly and Minton (1996) who documented RecA-independent genome restitution postirradiation. They reported that approximately 30% of the R1 genome is assembled in a *recA* background during the first 1.5 h after exposure, and they suggested that this process was single-stranded annealing. The DdrA protein could act directly or indirectly in any single-stranded annealing process that might occur in *Deinococcus.* Although the related Rad52 protein possesses a single-stranded annealing activity (Mortensen et al. 1996; Sugiyama et al. 1998), we have thus far failed to detect such an activity with DdrA protein. One of three explanations seems likely: (i) we have not yet identified suitable conditions for the assay of DdrA-dependent DNA strand annealing; (ii) DdrA is part of a complex, and other proteins are needed to observe activity; or (iii) DdrA does not possess such an activity.

DdrA's capacity to protect the 3′ ends of single-stranded DNA from digestion should help maintain the integrity of DNA fragments generated following DNA damage, whether those fragments are a result of the direct action of the damaging agent or arise as a consequence of a repair process that cleaves the phosphodiester backbone. By limiting degradation, proteins that protect DNA ends should enhance DNA damage tolerance and cell survival; the stabilized fragments serve as a long-lived substrate for homologous recombination or single-stranded annealing. In other words, we suspect that the ability to preserve genetic information is one key to understanding DdrA function and, in a larger context, the DNA damage tolerance of this species. DNA binding proteins, such as DdrA, may be particularly important for surviving desiccation. Like ionizing radiation, the process of desiccation is inherently DNA damaging, introducing large numbers of DNA double-stranded breaks. Following an extended period of desiccation, broken DNA ends would presumably need to be protected to minimize loss of genetic information. We know of no precedent for an activity of this sort in bacteria, although its existence has been predicted at least once (Clark 1991). Bacteriophage are known to encode proteins (e.g., the gene 2 protein of T4 [Wang et al. 2000]) that prevent exonucleolytic digestion of their genomes during infection, and given its sequence similarity to other phage proteins, it is possible that D. radiodurans acquired DdrA from a phage during its evolution.

Since inactivation of DdrA reduces, but does not eliminate, the DNA damage resistance of *Deinococcus,* we suggest that other proteins with complementary functions, possibly designed to bind DNA ends with different structures, are also encoded by this species, and the protection provided by these proteins contributes significantly to DNA damage tolerance. By itself, DdrA protein does not enhance the radiation resistance of E. coli strains in which it has been expressed (L. Alice Simmons and J. Battista, unpublished data).

It seems likely that *D. radiodurans,* and other bacteria with similar capacities to survive high DNA damage loads, employs multiple systems to repair its DNA. The DNA end-protection system we have begun to explore may be supplemented by special genome architectures (Levin-Zaidman et al. 2003), traditional DNA repair systems (some with unusual properties [Kim and Cox 2002]), and perhaps novel enzymatic systems not previously examined. Although we have detected no apparent enzymatic activities in DdrA to augment its DNA binding function, further work is needed to determine if DdrA contributes to single-stranded annealing or other potential DNA repair pathways. Bound to 3′ DNA ends, DdrA would be at a focus of DNA repair activity once genome restitution was initiated. The evolutionary relationship of DdrA to Rad52 may also telegraph a facilitating role in other DNA repair processes.

## Materials and Methods

### 

#### Strains, growth conditions, and treatment

Strains and plasmids used in this study are described in Table 2. All genes are identified as described in the published genome sequence (http://www.tigr.org/tigr-scripts/CMR2/GenomePage3.spl?database=gdr). All strains derived from D. radiodurans were grown at 30 °C in TGY broth (0.5% tryptone, 0.3% yeast extract, and 0.1% glucose) or on TGY agar (1.5% agar). E. coli strains were grown in Luria-Bertani (LB) broth or on LB plates at 37 °C. Plasmids were routinely propagated in E. coli strain DH5αMCR. D. radiodurans cultures were evaluated for their ability to survive exposure to DNA-damaging agents in exponential growth (OD600 = 0.08 − 0.15, 5 × 10^6^ − 1 × 10^7^ cfu/ml). All cultures were treated at 25 °C. Gamma irradiation was conducted using a model 484R ^60^Co irradiator (J. L. Shepherd and Associates, San Fernando, California, United States) at a rate of 30 Gy/min. Resistance to mitomycin C was determined by adding 1 mg of mitomycin C (Sigma, St. Louis, Missouri, United States) to 1-ml broth cultures of the D. radiodurans strain. Aliquots of the treated culture were removed at one-half-hour intervals over the next 2 h, washed in 10 mM MgSO4, and plated on TGY agar to determine viability.

**Table 2:**
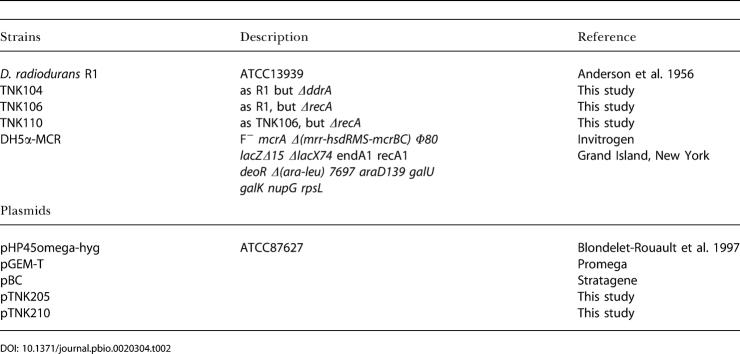
Strains and Plasmids

#### Construction of TNK104, TNK106, and TNK110

The genes *ddrA* and *recA* were disrupted by targeted mutagenesis using techniques described previously (Funayama et al. 1999). A deletion cassette was created for each locus and transformed into an exponential-phase D. radiodurans R1 culture. Recombinants were selected on TGY plates containing an appropriate antibiotic. Since D. radiodurans is multigenomic, individual colonies were screened to determine if they were homozygous for the disruption by isolating genomic DNA from putative recombinants and using a PCR-based analysis to determine whether the gene of interest had been deleted. Details for how each strain was generated are given below.

The construction of TNK104 began with the creation of a drug cassette capable of conferring hygromycin resistance on *D. radiodurans.* The hygromycin B phosphotransferase gene *(hyg)* from pHP45omega-hyg (Blondelet-Rouault et al. 1997) was spliced to the 120 bp of sequence immediately upstream of the initiation codon of the *D. radiodurans katA* gene (DR1998) (Funayama et al. 1999), using primers whose sequences overlapped. Subsequently, the *katA–hyg* fusion product was joined to PCR fragments (Horton et al. 1989) derived from the sequence 1.0 kbp immediately upstream and 0.9 kbp immediately downstream of *ddrA*. This hybrid fragment was cloned into pGEM-T (Promega, Madison, Wisconsin, United States), creating pTNK205. pTNK205 was propagated in E. coli DH5α-MCR. The deletion of *ddrA* was accomplished by transforming (Earl et al. 2002b) an exponential-phase R1 culture with linear pTNK205. Hygromycin-resistant recombinants were selected on TGY plates containing 37.5 μg/ml hygromycin.

To confirm gene replacement, primers, which anneal outside the coding sequence of *ddrA,* were used to generate PCR fragments from genomic DNA from hygromycin-resistant colonies and R1. The purified PCR products were restricted with EcoRI and EcoRV. The *hyg* gene contains an EcoRI site, but *ddrA* does not. *ddrA* contains an EcoRV site, but *hyg* does not. In the recombinant, designated TNK104, a single 1.3-kbp fragment, corresponding to the *katA–hyg* cassette was amplified, whereas there was no trace of the 0.85-kbp fragment, indicative of *ddrA* amplification (see Figure 1A). EcoRI cleaved the product amplified from TNK104 into 0.2-kbp and 1.1-kbp fragments, while the R1-derived product remained intact (Figure 1B). EcoRV digested the amplicon from R1 into fragments of 0.4 kbp and 0.45 kbp, but it did not affect the TNK104-derived product (Figure 1C). We conclude that TNK104 carries a deletion of the *ddrA* coding sequence marked by the *katA–hyg* cassette and that the strain is homozygous for the deletion.

The *recA* deletion strain TNK106 was constructed in a manner similar to that of TNK104. Initially, the *katA* promoter of D. radiodurans was fused to the chloramphenicol acetyltransferase gene *(cat)* from pBC (Stratagene, La Jolla, California, United States). This drug cassette was then spliced to PCR products corresponding to genomic DNA sequences 1.6 kbp upstream and 1.2 kbp downstream of *recA* by overlap extension, before being cloned into pGEM-T. The resulting plasmid was designated pTNK210. An exponential-phase R1 culture was transformed with the replacement cassette from pTNK210, and chloramphenicol-resistant recombinants were selected on TGY plates containing 3 μg/ml chloramphenicol. Genomic DNA of each recombinant was amplified to determine if the *recA* coding region was deleted. Purified PCR products amplified using primers that anneal to sequences flanking *recA* were treated with PvuII and BglII. The *cat* gene carries a PvuII site, but *recA* does not. *recA* contains a BglII site, but *cat* does not. A 1.3-kbp fragment, corresponding to the *katA–cat* cassette, was obtained from a recombinant designated TNK106, but DNA from this recombinant did not generate the 1.5-kbp fragment corresponding to *recA* (Figure 1A). Amplifications of genomic DNA from R1 only produced the 1.5-kbp fragments (Figure 1A). The 1.3-kbp PCR product from TNK106 was cleaved by PvuII to 0.5-kbp and 0.8-kbp fragments, whereas the 1.5 kbp from R1 remained intact (Figure 1C). BglII cut the R1-derived 1.5 kbp to fragments of 0.45 kbp and 1.05 kbp, but not the product from TNK106 (Figure 1C). We conclude that *recA* has been replaced by *katA–cat* in TNK106 and that the strain is homozygous for this allele. TNK110 is a double mutant in which *recA* and *ddrA* are deleted. This strain was constructed by deleting *recA* from TNK104 using the protocol described for the creation of TNK106. The construct was verified by the scheme used to identify *ddrA* deletion in TNK104 and *recA* deletion in TNK106 (see Figures 1A and 1C).

#### Pulsed-field gel electrophoresis

After irradiation at 5.0 kGy, cells were collected by centrifugation (6,000*g,* 15 min, 4 °C) and resuspended in either TGY broth or 10 mM MgSO4 solution, before being placed in a shaking incubator at 30 °C for 24 h. Aliquots of these cultures were removed at various time points, and cells were washed in 0.9% NaCl and suspended in 0.125 M EDTA (pH 8.0) at a density of 5 × 10^8^ cells/ml. The suspensions were mixed with low-melting-point agarose (Sigma) to obtain a final concentration of 0.8% agarose. Agarose blocks containing the cell suspension were incubated overnight at 37 °C in 0.05 M EDTA (pH 7.5) containing 1 mg/ml of lysozyme. After lysozyme treatment, agarose plugs were placed in ESP buffer (EDTA 0.5 M [pH 9–9.5], 1% lauroyl sarcosine, 1 mg/ml proteinase K) at 50 °C for 6 h, followed by a 2-d incubation at 37 °C. Prior to digestion with restriction enzymes, agarose plugs were washed once with TE buffer (pH 7.5) containing 1 mM phenylmethylsulfonyl fluoride and then four times with TE buffer (pH 7.5). DNA contained within the agarose plugs was digested with 10 U of NotI restriction enzyme (New England Biolabs, Beverly, Massachusetts, United States) overnight at 37 °C. Restriction digests were analyzed on 1% agarose gels in 0.5X TBE, using a CHEF-MAPPER electrophoresis system (Bio-Rad, Hercules, California, United States) at 6 V/cm for 22 h at 12 °C, with a linear pulse ramp of 10–60 s and a switching angle of 120°. Gels were stained with water containing 0.5 μg/ml ethidium bromide for 20 min and destained for 10 min in water.

#### Quantitative real-time PCR

The protocol followed was the same as that described previously (Earl et al. 2002a). Total RNA was extracted from 1-l cultures of irradiated and nonirradiated exponential-phase D. radiodurans cultures using TRI Reagent (Molecular Research Center, Cincinnati, Ohio, United States) following manufacturer's instructions. Cell disruption was accomplished by adding 100 μ1 of 0.1-mm zirconia/silica beads (Biospec Products, Bartlesville, Oklahoma, United States) and TRI Reagent to the cell paste from 1 l of cells and vigorously agitating this mixture for 6 min with a vortex mixer. Two micrograms of each DNase I–treated, purified RNA sample was converted to cDNA using SUPERSCRIPT II RNase H^−^ Reverse Transcriptase (Invitrogen, Carlsbad, California, United States) combined with 25 pmol of random hexamers to initiate synthesis. Conditions for this reaction followed the manufacturer's instructions.

Approximately 100 bp of unique sequence from the genes encoding DdrA (DR0423), RecA (DR2340), and glyceraldehyde 3-phosphate dehydrogenase (DR1343) were amplified using the following primer sets: DR0423up (5′-GGTGCAGGACCGACTCGACGCCGTTTGCC-3′), DR0423down (5′-CCTCGCGGGTCACGCCGAGCACGGTCAGG-3′), DR2340up (5′-GTCAGCACCGGCAGCCTCAGCCTTGACCTC-3′), DR2340down (5′-GATGGCGAGGGCCAGGGTGGTCTTGC-3′), and DR1343up (5′-CTTCACCAGCCGCGAAGGGGCCTCCAAGC-3′), DR1343down (5′-GCCCAGCACGATGGAGAAGTCCTCGCC-3′). The PCR reaction (50 μ1) for amplifying these genes contained the appropriate primers at a final concentration of 0.2 μM, 1 μ1 of the cDNA template, and SYBR Green PCR Core Reagents (Applied Biosystems, Foster City, California, United States). Amplifications were carried out by incubating reactions at 95 °C for 3 min prior to 40 cycles of 30 s at 95 °C, followed by 30 s at 65 °C and 30 s at 72 °C. Data were collected and analyzed at each 72-°C interval. Each 96-well plate consisted of standard curves for each primer set run in duplicate. Standard curves were constructed using cDNA obtained from the unirradiated wild-type organism. A dilution series (1 to 1 × 10^−4^) of each experimental sample was generated and run in duplicate. Negative controls without a cDNA template were run on every plate analyzed. All assays were performed using the iCycler iQ Real-Time Detection System (Bio-Rad). All data were PCR-baseline subtracted before threshold cycle values were designated and before standard curves were constructed. Mean concentrations of the transcripts in each sample were calculated from the standard curves generated using the *recA* primer set. Induction levels were determined by dividing the calculated concentration of transcript from the irradiated sample by the concentration of transcript from the unirradiated sample for each strain. The mean concentration of the *gap* transcript, a housekeeping gene whose expression is unaffected by ionizing radiation, was also determined before and after irradiation for each strain.

#### DNA content measurement in TNK104 and R1 cells

Overnight cultures growing in TGY medium were harvested at room temperature. Control culture aliquots were fixed with 1% toluene (final vol/vol), shaken vigorously, and stored at 4 °C. The fixed bacteria were diluted (1/10, 1/100, and 1/1,000) in 3 ml (final volume) of dilution buffer: 10 mM NaCl, 6.6 mM Na_2_SO_4_, 5 mM N′-2-hydroxyethylpiperazine-N′-2-ethanesulfonic acid (HEPES; pH 7.0). The remaining cultures were centrifuged for 20 min at 4 °C at 7,000 rpm. Bacterial pellets were washed twice and resuspended in 10 mM MgSO_4_ for γ irradiation. Cell suspensions were irradiated at 5,000 Gy and incubated at 30 °C for 120 h. Aliquots were removed immediately following irradiation, at 48 h, and at 120 h postirradiation. Cells were toluene-fixed as described above; 100 μ1 of DAPI (stock solution 3 μg/ml) was added to each dilution tube and mixed. The fluorescence intensity was determined after excitation at 350 nm by measuring emission at 450 nm.

#### Cloning, overexpression, and purification of DdrA

The *ddrA* gene was amplified using the genomic DNA from D. radiodurans strain R1. PCR primers were designed according to the *ddrA* gene sequence annotated in the genomic bank (http://www.ncbi.nlm.nih.gov). The gene was cloned in E. coli overexpressing plasmid pEAW298. DdrA-overproducing cells were lysed with lysozyme, and the protein was precipitated from the supernatant by adding ammonium sulfate to 30% saturation. The protein was purified with DEAE and hydroxyapatite chromatography to greater than 99% purity. The identity of the purified protein was confirmed by N-terminal sequencing (Protein and Nucleic Acid Chemistry Laboratory, Washington University School of Medicine, St. Louis, Missouri, United States) and accurate mass determination (Biotech Center, University of Wisconsin, Madison, Wisconsin, United States). The protein was transferred into the storage buffer (20 mM Tris-acetate, 80% cation [pH 7.5]/50% glycerol [w/v], 0.5 M NaCl, 0.1 mM EDTA, and 1 mM DTT) and stored at −80 °C.

#### Determination of the extinction coefficient for pure DdrA protein

The extinction coefficient for DdrA protein was determined using a modification of a published procedure (Marrione and Cox 1995). UV absorbance spectra were measured with a Cary 300 dual-beam spectrophotometer (Varian, Palo Alto, California, United States). The temperature was maintained using a circulating water bath. Cell-path length and bandwidth were 1 cm and 0.5 nm, respectively. The extinction coefficient for native DdrA protein was determined in the storage buffer, by comparing the absorbance spectra of the native protein to the absorbance spectra of the protein denatured in 6 M guanidine hydrochloride (Gnd–HCl) in storage buffer. The extinction coefficients at 280 nm of glycyl-L-tyrosylglycine and *N*-acetyl-L-tryptophanamide in 6 M GND–HCl are 1,280 M^−1^cm^−1^ and 5,690 M^−1^cm^−1^, respectively (Edelhoch 1948). In the DdrA protein there are five tyrosine, five tryptophan, and two cysteine residues in a protein with a total molecular mass of 23 kDa. Even if all cysteine residues were involved in disulfide bonds, the contribution of cystine to the absorbance of DdrA protein is predicted to be less than 1% and was neglected from our calculations. The extinction coefficient at 280 nm for denatured DdrA protein in ɛ_denat, 280 nm_ = 5 × 5,690 + 5 × 1,280 = 3.485 × 10^4^ M^−1^cm^−1^. Absorbance spectra of native and denatured (6 M GND–HCl) DdrA protein were scanned at 25 °C, from 320 to 240 nm, for five different dilutions and with two different protein preparations. DdrA protein was diluted in storage buffer or storage buffer plus 6 M GND–HCl (final concentration) in a total volume of 80 μ1 and was preincubated at 25 °C for 5 min before scanning. Each dilution was carried out in triplicate, and the absorbance values at 280 nm were averaged. The concentrations of native and denatured protein were equal to each other in each scan at each dilution. The extinction coefficient of native DdrA protein at 280 nm was determined according to the expression (Gill and von Hippel 1989): ɛ_nat, 280 nm_ = ɛ_denat, 280 nm_ × Abs_nat, 280 nm_/Abs_denat, 280 nm_. We used five determinations with two different protein preparations, yielding an average extinction coefficient of ɛ_nat, 280 nm_ = 2.8728 ± 0.1999 × 10^4^ M^−1^cm^−1^ in storage buffer at 25 °C. The A_280_/A_260_ ratio for the native DdrA protein is 1.575 ± 0.00091. The error in both cases is 1 s.d.

#### DNA binding assay

The duplex oligonucleotide with a 3′ single-stranded extension was hairpin-forming oligonucleotide A (5′-TTA ACG ACC GTC GAC CTG CAG GTC GAC GGT CGT TAA CGT CTC TCA GAT TGT-3′), which was labeled at the 3′ terminus with [α-^32^P]ddATP, using terminal transferase. After labeling, hairpin formation generated an 18-bp duplex hairpin with a 16-nt 3′ extension. The duplex oligonucleotide with a 5′ single-stranded extension was hairpin-forming oligonucleotide B (5′-CGT CTC TCA GAT TGT TTA ACG ACC GTC GAC CTG CAG GTC GAC GGT CGT TAA-3′). The oligo was labeled at the 5′ end using [γ-^32^P] ATP and polynucleotide kinase. After labeling, hairpin formation generated a DNA with 18 bp in the hairpin duplex and a 15-nt 5′ extension. A blunt-ended duplex DNA fragment was prepared by annealing oligonucleotide C (5′-GGT CTT TCA AAT TGT TTA AGG AAG AAA CTA ATG CTA GCC ACG GTC CGA GCC-3′) ^32^P-labeled at its 5′ end, with unlabeled oligonucleotide D (5′-GGC TCG GAC CGT GGC TAG CAT TAG TTT CTT CCT TAA ACA ATT TGA AAG ACC-3′). The single-stranded oligonucleotide was the end-labeled oligo C. EMSAs for DNA binding were carried out in 15-μl reaction mixtures containing the reaction buffer (40 mM Tris-acetate [pH 7.5],10% glycerol [w/v], 0.1 M NaCl, 0.1 mM EDTA, 1 mM DTT) and 0.7 nM (60 nM nt) ^32^P-labeled duplex DNA. The reaction was initiated by adding the DdrA protein to the required concentration. The reaction mixture was incubated at 30 °C for 30 min and loaded onto a 10% native polyacrylamide gel. The electrophoresis was performed in 1X TBE (89 mM Tris-borate [pH8.3], 2 mM EDTA) at room temperature. After the electrophoresis was completed, the gel was dried and exposed with a Phosphoimager (Molecular Dynamics, Sunnyvale, California, United States).

#### Identification of DdrA protein in DNA–protein complex

The general strategy of this experiment was to incubate a DNA duplex with a 3′ extension with DdrA protein, resolve the protein complex in native PAGE, excise the complex from the gel, extract the protein from the slice, and analyze the protein in SDS-PAGE. If the protein is DdrA, it will comigrate with DdrA protein in SDS-PAGE.

A ^32^P-labeled oligonucleotide (30 nt; 5′-GTG CGC TCC GAG CTC AGC TAC CGC GAG GCC-3′) was annealed with a longer unlabeled oligonucleotide (50 nt; 5′-GGC CTC GCG GTA GCT GAG CTC GGA GCG CAC GAT TCG CAC TGC TGA TGT TC-3′). Annealing was carried out in a 40-μ1 solution containing 0.5 μM of each oligonucleotide in 25 mM Tris HCl (pH 8), 50 mM NaCl, and 12.5 mM MgC1_2_. The solution was heated briefly at 100 °C, by transferring the closed tube to a beaker of boiling water, and allowed to cool slowly overnight. The tube was refrigerated for several hours and then stored at −20 °C until use.

The resulting labeled duplex DNA with a 3′ extension (0.7 nM) was incubated with 4 μM DdrA protein under the DNA binding conditions described above. The mixture was loaded onto a 10% native polyacrylamide gel. Electrophoresis was performed as described above. The gel was exposed with X-ray film to map the position of the protein–duplex complex. The complex was cut out of the gel. The gel slice was frozen in liquid nitrogen and crushed into a slurry with a plastic stick. The slurry was mixed with an equal volume of SDS-PAGE loading buffer and boiled for 3 min. The mixture was loaded onto a 12% SDS-PAGE gel and the protein present compared to molecular weight standards and purified DdrA protein.

#### Exonuclease assay

The duplex with a 3′ extension was prepared by annealing oligonucleotide A (5′-CTA GCA TTA GTT TCT TCC TTA AAC AAT TTG AAA GAC C-3′), which was labeled at the 5′ terminus with [γ-^32^P]ATP, and cold oligonucleotide B (5′-GGT CTT TCA AAT TGT TTA AGG AAG AAA CTA ATG CTA GCC ACG GTC CGA GCC-3′). The annealing generated a 14-nt 3′ extension at one end of the short duplex. Before adding the exonuclease, the ^32^P-labeled duplex (60 nM nt) was preincubated with the DdrA protein at the indicated concentration in 15 μl of the exonuclease reaction buffer (40 mM Tris-acetate [pH 7.5], 0.1 M NaCl, 10 mM MgC12, 0.1 mM EDTA, 1 mM DTT, 10% glycerol) at room temperature for 10 min. In the control experiment, the DdrA protein was replaced with bovine serum albumin. Exonuclease I was added to 200 U/ml and the reaction mixture was incubated at 37 °C for 30 min. After the incubation was complete, the reactions 5 and 6 were deproteinized with 0.2% SDS and 0.2 mg/ml proteinase K at 37 °C for 15 min. The DNA–protein complexes were resolved in the native polyacrylamide gel as above.

## Supporting Information

### Accession Numbers

The GenBank (http://www.ncbi.nlm.nih.gov/Genbank/index.html) accession numbers for the genes and gene products discussed in this paper are *ddrA*/DR0423 (NP_294146), Erf (P04892), *gap/*DR1343 (NP_295066), Rad52 (P06778), *recA/*DR2340 (NP_296061), RecT (P33228), and Redβ (P03698).

## Abbreviations

cfu: colony-forming unit
*ddrA*: DNA damage response A gene
DdrA: DNA damage response A protein
EMSA: electrophoretic mobility-shift assay
GND–HCl: guanidine hydrochloride
nt: nucleotide
SDS: sodium dodecyl sulfate
