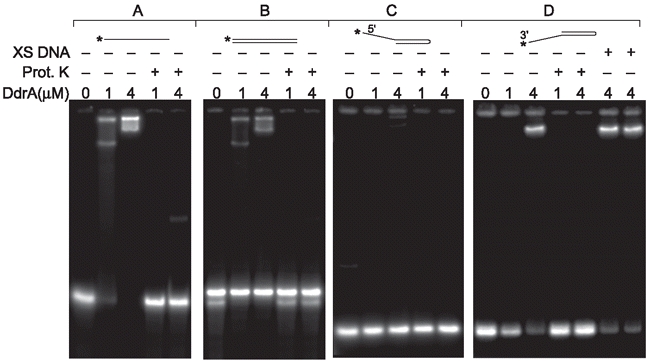# Correction: Preserving Genome Integrity: The DdrA Protein of Deinococcus radiodurans R1

**DOI:** 10.1371/journal.pbio.0040385

**Published:** 2006-11-14

**Authors:** Dennis R Harris, Masashi Tanaka, Sergei V Saveliev, Edmond Jolivet, Ashlee M Earl, Michael M Cox, John R Battista

In *PLoS Biology*, volume 2, issue 10: DOI: 10.1371/journal.pbio.0020304

There was an error in Figure 7D. Proteinase K was not added to reactions with XS DNA. Instead, the preceding two reaction lanes should have “+” symbols, indicating the addition of Proteinase K, as shown in the corrected Figure.